# The Double-Edged Sword Effect of Interaction Frequency with AI on College Students: The Moderating Role of Peer Support

**DOI:** 10.3390/bs15091267

**Published:** 2025-09-16

**Authors:** Wenyan Sun, Zhanfeng Jiang, Shenyang Hai

**Affiliations:** 1 School of MarXism, Henan Normal University, Xinxiang 453000, China; sunweny320@163.com; 2 International Business School, Hainan University, Haikou 570100, China

**Keywords:** artificial intelligence, need for affiliation, loneliness, problematic mobile phone use, prosocial behavior, peer support

## Abstract

Generative artificial intelligence (AI) has become an indispensable resource in contemporary higher education, providing substantial benefits to both students and institutions. As its adoption accelerates, it is important to balance these advantages against potential risks that may arise from students’ varying levels of interaction with AI. Whereas most prior studies have focused on the favorable outcomes of AI for college students, the present research investigates its “double-edged sword” effects. Guided by social affiliation theory, a new model has been developed and empirically tested to clarify how and under what conditions the frequency of student–AI interaction influences social needs and behaviors. Longitudinal data obtained from 388 undergraduates showed that AI interaction frequency can shape prosocial behavior and problematic mobile phone use through a dual pathway involving the need for affiliation and feelings of loneliness. Furthermore, peer support moderates the indirect effect of AI interaction frequency on college students’ prosocial behavior via their need for affiliation. The results showed that peer support did not significantly moderate the indirect relationship between AI interaction frequency and problematic mobile phone use through loneliness. Overall, our study extends the framework of the social affiliation theory and provides practical insights that guide the appropriate use of AI by college students, thereby supporting the development of healthy social skills and technology engagement in the age of AI.

## 1. Introduction

With the rapid advancement of artificial intelligence technology across educational domains, AI adoption among university students is experiencing unprecedented growth (Crompton & Burke, 2023). However, beneath this rapid ubiquity, emerging research reveals a concerning phenomenon. While AI has been productively applied to content adaptation and personalized tutoring (Lin et al., 2023), and has even shown benefits in improving emotional well-being through assisted venting (Hu et al., 2025), it may also lead to potential risks such as increased loneliness (Crawford et al., 2024), over-dependence (Zhang et al., 2024), and declining social skills (Eairheart & Azimzadeh, 2025). The relationship between university students and AI may therefore be more complex than anticipated.

Based on a literature review of research on university students’ AI use, on one hand, some studies suggest that AI can promote individual social skills by providing personalized learning opportunities (Bhutoria, 2022); on the other hand, other studies propose that over-reliance on AI may lead university students to lack authentic interactions with peers (Crawford et al., 2024), thereby hindering social relationship development. To better explain these inconsistent findings, it is necessary to integrate a comprehensive theoretical framework to elucidate the mechanisms by which AI usage affects university students’ social–psychological development.

In research examining the impact of AI interactions on university students, a critical yet underexplored dimension is the role of interaction frequency. Traditionally, university students relied more on interactions with teachers and classmates, but many decision-making and learning problems are now directly addressed to AI (Seo et al., 2021). Specifically, AI’s characteristics of immediate feedback and support have led to a substantial increase in university students’ frequency of AI interactions, and artificial intelligence interaction frequency therefore warrants attention.

AI interaction frequency refers to the degree of individual participation, usage, or interaction with AI systems during task processes, reflecting university students’ transition from traditional interpersonal interaction toward human–AI hybrid interaction models in the digital age. Unlike traditional technology usage variables (such as usage duration and functional preferences), AI interaction frequency captures the dynamic interactive process between university students and intelligent systems. This interaction possesses quasi-social properties. While AI itself does not possess a sense of social belonging, it can, to some extent, evoke or satisfy human users’ need for social belonging and emotional support through its functions and interactive methods. AI is capable of conducting fluent conversations, understanding and responding to human language, and even mimicking human tone and expression. This makes human–AI dialog sound more like human-to-human interaction (Ho et al., 2025). This poses a pivotal question: In contrast to conventional interpersonal interactions, how do these human–AI interaction mechanisms affect the internal psychological processes of college students?

To explore this question in depth, this study draws upon social affiliation theory as the core theoretical framework. Proposed by O’Connor and Rosenblood (1996), the theory posits that humans possess an innate basic psychological need for social connection and maintain an internal system that automatically monitors social quality. When individuals detect social insufficiency, instinctual driving mechanisms are activated, prompting them to seek compensatory behaviors to restore social connections. Compared to other possible compensatory behaviors (such as social skills and interpersonal trust), the need for affiliation represents individuals’ fundamental desire for social connection and serves as the primary target of the social monitoring system (Cherbuin et al., 2021), reflecting the ideal expectations of “desired states”. Loneliness is the subjective experience generated when the social monitoring system detects social insufficiency, serving as a direct signal of unmet social needs and reflecting the realistic predicament of “deficient states”. This phenomenon has been empirically demonstrated in emerging research; for example, the frequency of AI interaction can trigger compensatory mechanisms (Tang et al., 2023). We propose that a social evaluation system composed of these two variables (need for affiliation; loneliness) may offer a more comprehensive framework for capturing the impact of AI interaction frequency on psychological states, moving beyond previous research that often relied on single mediating variables.

Peer support may serve as a moderating function in this process. From the human–computer interaction research perspective, peer support represents the quality of authentic social feedback (Smith et al., 2021). High levels of peer support may mitigate the negative effects of missing social feedback in AI interactions, while low levels of peer support may amplify such effects. Social affiliation theory indicates that peer support directly influences the social monitoring system’s assessment of current social conditions. When individuals perceive sufficient peer support, even frequent AI interactions are unlikely to trigger strong signals of social insufficiency (Daou et al., 2020). Conversely, under conditions of insufficient peer support, AI interactions may exacerbate individuals’ sensitivity to social deficits, leading to heightened need for affiliation and loneliness. Peer support, as a key social contextual factor, therefore moderates the intensity and direction of the impact AI interaction frequency has on university students’ internal psychological states.

**Figure 1:**
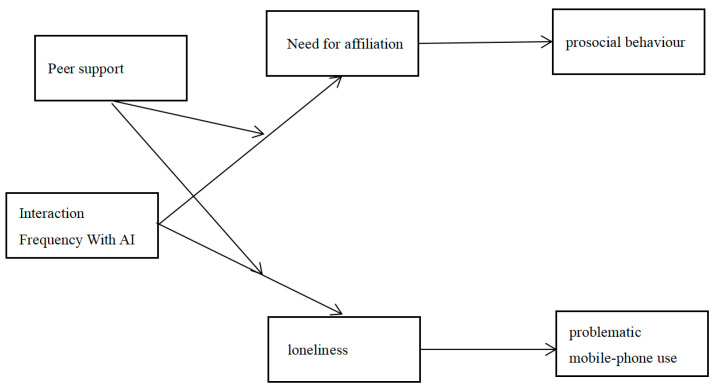
Hypothesis model for the double-edged sword effect of interaction frequency with AI on college students: the moderating role of peer support.

This study draws upon two theoretical perspectives. The first perspective derives from human–computer interaction research, suggesting that AI’s lack of authentic social feedback may lead university students to urgently seek interpersonal connections or experience loneliness. The second perspective is grounded in social affiliation theory (O’Connor & Rosenblood, 1996), which emphasizes that humans possess an internal system for monitoring social quality and activating compensatory mechanisms when social insufficiency is detected. This study integrates these theoretical perspectives to construct and empirically test a model examining the impact of AI interaction frequency on university students’ need for affiliation, loneliness, and behavioral outcomes (see Figure 1). In response to the question, “How does AI interaction frequency influence college students’ adaptive behaviors through the mediating roles of the need for affiliation and loneliness, and how is this pathway moderated through peer support?”, we propose the following hypotheses: H1: AI interaction frequency will be positively associated with the need for affiliation; H2: The need for affiliation will be positively associated with prosocial behavior; H3: AI interaction frequency will be positively associated with problematic mobile phone use; H4: Loneliness will be positively associated with problematic mobile phone use; H5: Peer support will weaken the positive effect of AI interaction frequency on the need for affiliation; H6: Peer support will weaken the positive effect of AI interaction frequency on loneliness.

This study makes significant contributions to the literature examining the impact of AI interaction frequency on university student development, aiming to promote university students’ enhancement of social skills and achievement of social developmental maturation in the context of increasingly anthropomorphic AI. This study contributes significantly in three key domains. First, it systematically extends social affiliation theory to AI interaction contexts, establishing an “AI interaction–need for affiliation/loneliness behavioral outcome” theoretical framework that fills the application gap of traditional theory in the digital age. Second, by introducing peer support as a moderating variable, it reveals the boundary conditions of AI interaction effects. Under different levels of social support, the intensity and direction of AI interaction’s impact on psychological needs and behavioral outcomes undergo changes, thereby enriching the predictive and explanatory power of the theory. Third, by targeting university student populations in social transition periods, it reveals how they seek balance between authentic social interaction (peer support) and virtual social interaction (AI interaction), and how this balance influences psychological states and behavioral performance. Finally, this study provides multi-level intervention strategies for universities’ mental health initiatives. Regarding concerns about the impact of university students’ AI usage on social relationships, interventions can directly influence students’ psychological needs by modulating AI interaction frequency or optimizing the positive effects of AI interaction by enhancing peer support.

### 1.1. College Students and AI Interaction Frequency

The rapid development of artificial intelligence technology is profoundly reshaping higher education ecosystems, particularly redefining university students’ learning processes and interaction modalities (Velastegui et al., 2023). AI interaction has demonstrated beneficial outcomes in this context. A meta-analysis revealed that the novelty effect of AI chatbots in short-term interventions can significantly enhance learning outcomes (Wu & Yu, 2024). Students can leverage AI as a conversational assistant, fostering a sense of security and connection through moderate AI interaction, thereby mitigating negative emotions and psychological distress (Maples et al., 2024). However, scholars have expressed concerns regarding the efficacy of high-frequency AI interaction—as interaction frequency increases, detrimental effects begin to manifest. Frequent human–AI interaction may trigger cognitive offloading (Gerlich, 2025), a phenomenon where individuals transfer excessive cognitive burdens to AI systems, progressively attenuating critical thinking, memory, and decision-making skills over time and with increased interaction frequency. Furthermore, AI interaction can exacerbate psychosocial risks. Systematic research indicates that university students who excessively rely on conversational systems embedded with generative models face risks such as diminished decision-making, critical thinking, and analytical reasoning abilities. When factors such as social support, psychological well-being, loneliness, and sense of belonging are holistically considered, high-frequency AI interaction exerts a net negative impact on academic achievement (Crawford et al., 2024). These findings suggest that AI interaction frequency not only influences learning and cognition but also more profoundly addresses fundamental issues concerning human social needs. Bridging back to our study context, the increasing augmentation of college students with AI upends this traditional social structure (interpersonal interactions) by coupling college students with intelligent machines with whom they also interact (Wilson & Daugherty, 2018). It is therefore crucial to investigate how these human–AI interaction experiences influence university students’ social adaptability and behavioral performance.

This study draws upon but simultaneously challenges the core assumptions of traditional social affiliation models (O’Connor & Rosenblood, 1996). The social affiliation model posits that humans possess an innate basic need for social connection and maintain an internal system for automatically monitoring social quality. When social insufficiency is detected, instinctual driving mechanisms are activated, prompting individuals to seek compensatory behaviors. However, this theory was established based on traditional face-to-face interpersonal interaction contexts, and it may yield novel outcomes in human–computer interaction contexts; for example, contemporary Chinese university students tend to position AI as collaborative partners for learning and decision-making. Since AI lacks authentic emotional feedback and nonverbal signals (Barrett et al., 2019), it may trigger individuals’ internal social quality monitoring systems, leading to increased need for affiliation and loneliness. Unlike the single positive compensation expected by traditional theory, to address this social threat, university students’ instinctual drives may activate two distinctly different compensatory strategies. On one hand, there is active pursuit of authentic social compensation (such as prosocial behavior), and on the other hand, passive withdrawal (such as problematic mobile phone use). Prosocial behavior and problematic smartphone use represent two “co-occurring outcomes” stemming from university students’ frequent interactions with artificial intelligence.

This dual-pathway model reveals unique psychological paradoxical phenomena in AI interactions, constituting an important extension of traditional social affiliation theory. The directionality of such compensatory behaviors may be moderated through individuals’ actual social environments. To clarify this theoretical boundary condition, based on the collectivistic cultural characteristics and high peer dependency of Chinese university student populations, this study identifies peer support as a key contextual factor influencing the effects of university students’ AI interactions.

### 1.2. AI Interaction Frequency, Need for Affiliation, and Prosocial Behavior

Numerous studies indicate that university students increasingly view AI as learning partners or collaborators rather than merely instrumental applications. Particularly in post-pandemic hybrid learning models, human–computer interaction has shifted from sporadic use to high-frequency, routine collaborative patterns. Students’ prolonged and frequent interactions with AI in this process may therefore engender a novel form of social connection.

There is an increasing body of literature confirming that humans require social interaction to thrive and survive. According to the social affiliation theoretical framework proposed by O’Connor and Rosenblood (1996), humans possess an intrinsic regulatory process for continuously monitoring the quality of their social relationships with others. Baumeister and Leary’s (1995) belongingness theory, along with other related work, serves as part of the broader “social affiliation literature” that informs. The fulfillment of belongingness needs is not exclusively confined to interpersonal relationships but can be achieved through diverse forms of social interaction. This system evaluates individuals’ current level of social integration by interpreting verbal and nonverbal signals in social interactions. When university students perceive low levels of social connection, this regulatory process activates “social driving mechanisms,” prompting individuals to take corrective action. However, in the contemporary Chinese higher education environment (Yan et al., 2025), how does human–AI collaboration influence individual social belonging need-satisfaction mechanisms?

Artificial intelligence is often viewed as a supplementary learning tool in educational settings, and this perspective is also supported by empirical research in the West, e.g., on chatbots and simulated instructors in interactive learning platforms (Y. F. Lee et al., 2022; Golding et al., 2025; Baek et al., 2024). Through high-frequency interactions with AI, university students may perceive these AI systems as social interaction partners to some extent.

Returning to this study’s context, the widespread application of AI technology in university students’ learning processes may disrupt this traditional social regulatory mechanism. Specifically, AI systems fundamentally lack the capacity for nuanced nonverbal communication elements in interpersonal interactions, such as facial expressions, eye contact, and body posture (Wirtz et al., 2019). Furthermore, these technological interfaces demonstrate significant limitations in authentic emotional expression and empathetic responses (Nomura et al., 2006), resulting in the absence of meaningful social signal transmission during student–AI interactions. Simultaneously, most contemporary AI systems operate using “standardized, mechanistic interaction patterns” (Wirtz et al., 2019), which sharply contrast with university students’ preferences for personalized, emotionally resonant interpersonal communication needs (Moeller et al., 2020).

According to the perspective of O’Connor and Rosenblood’s (1996) social affiliation model, when university students frequently engage with AI systems that are socially perceived and interpret the standardized, non-empathetic responses of these AI systems as cues of social rejection, this persistent, depersonalized interaction thus engenders a notable discrepancy between their desired level of social connection and the actual outcomes experienced (Crawford et al., 2024). This directly activates a heightened need for social relatedness, consequently fostering a strong motivation and desire among university students to engage in human interaction, aimed at compensating for the perceived deficit in social connection arising from AI interactions. A higher frequency of interaction may therefore lead to a greater need for affiliation.

**Hypothesis** **1.** *AI interaction frequency will be positively associated with the need for affiliation.*

Based on the compensatory motivation perspective of social affiliation theory, this study further proposes the following hypothesis: When university students experience social deprivation during human–computer interactions, which triggers stronger need for affiliation, instinctual driving mechanisms will prompt them to seek social contact as a compensatory means (O’Connor & Rosenblood, 1996). Specifically, when individuals encounter social deprivation states, their physiological and psychological systems automatically activate social compensation mechanisms, driving individuals to actively seek social connections to maintain appropriate levels of social interaction. To prevent the negative effects of social isolation (Lim et al., 2022), individuals need to maintain a certain degree of social interaction frequency. In this context, prosocial behavior, particularly providing supportive assistance to others in campus environments, serves as a low-cost, high-efficacy social connection strategy, offering a viable compensatory pathway for individuals experiencing belonging deficits (Walton et al., 2023). In the collectivistic cultural atmosphere of Chinese university campuses, this compensatory pathway may be more effective, as helping behaviors are often viewed as important indicators of personal virtue and social responsibility.

For individuals with a high need for affiliation and current belonging deficits, prosocial behavior exhibits characteristics of low risk and high visibility, making it an ideal social connection compensation mechanism. Compared to other social behaviors, prosocial behavior (such as helping behavior) is typically viewed by recipients as positive behavior, with a relatively low probability of encountering rejection or negative evaluation (Chávez et al., 2022). Furthermore, prosocial behavior possesses obvious social signaling functions, capable of transmitting positive personal quality information to potential social partners, thereby facilitating the establishment of subsequent social relationships. Based on these characteristics, university students’ engagement in prosocial behaviors such as helping classmates, participating in group activities, and providing academic support becomes an effective avenue for satisfying the need for affiliation. This behavioral pattern not only satisfies individuals’ social affiliation needs but also provides effective pathways for constructing and maintaining interpersonal relationship networks. Prosocial behavior can therefore be viewed as an adaptive means for individuals to cope with social deprivation states and restore social relationship balance. In contemporary contexts where Chinese university students face increased AI interactions while experiencing relatively decreased authentic social interactions, the role of this compensatory mechanism may become more pronounced.

**Hypothesis** **2.** *The need for affiliation will be positively associated with prosocial behavior.*

### 1.3. AI Interaction Frequency, Loneliness, and Problematic Mobile Phone Use

Loneliness refers to “a passive, distressing emotional state triggered by the perceived gap between an individual’s desired level of social connection and the actual level obtained” (Cacioppo & Patrick, 2008). This concept emphasizes the subjective characteristics of loneliness, whereby its generative mechanism is based on individuals’ cognitive evaluation processes rather than objective social isolation states.

When individuals interact with AI, their social monitoring system automatically seeks out social feedback cues (e.g., emotional resonance, personalized responses, mutual understanding) (Tang et al., 2023). In human–computer interaction contexts, AI’s essential characteristics (standardized response patterns, lack of emotional authenticity, and depersonalized communication styles) render it incapable of providing key social relationship connections found in interpersonal interactions (such as emotional resonance, mutual understanding, and personalized attention). Indeed, the standardized responses of AI cannot provide the anticipated social feedback cues, thereby creating a persistent “expectation–reality” mismatch. This mismatch can lead to a continuous disappointment of social expectations, which is interpreted by the brain as “being ignored” or “not understood,” subsequently triggering loneliness (Crawford et al., 2024). According to the O’Connor and Rosenblood (1996) social affiliation model perspective, when individuals’ social monitoring systems continuously detect this state of “insufficient social relationship connection,” chronic social dissatisfaction may emerge, ultimately manifesting as the accumulation and intensification of lonely emotions. A growing body of research across diverse cultural contexts has consistently demonstrated the risk of loneliness associated with artificial intelligence use (Dong et al., 2025).

**Hypothesis** **3.** *AI interaction frequency will be positively associated with problematic mobile phone use.*

This study further hypothesizes that loneliness induced by AI interaction frequency promotes problematic smartphone use among university students. When individuals experience negative emotional states, they tend to adopt immediately available methods to alleviate psychological distress (Kardefelt-Winther, 2014). Mobile devices serve as ideal emotional avoidance tools due to their ubiquity and convenience. For example, adolescents frequently use smartphones to access social media platforms such as LINE to reduce feelings of loneliness.

Problematic smartphone use refers to excessive and uncontrollable mobile device usage that negatively impacts an individual’s daily life (Y. T. Hu & Wang, 2022). Existing research primarily focuses on factors influencing problematic smartphone use, including anxiety (Augner et al., 2023), parenting styles, and other emotional and environmental factors. However, among contemporary Chinese university students, frequent and uncontrolled problematic smartphone use may represent an important strategy for coping with loneliness. As a maladaptive coping mechanism, smartphones serve as common tools for alleviating loneliness among young adults due to their convenience and capacity for attention diversion and emotional numbing (Li et al., 2021). Specifically, when university students experience distress from AI interaction-induced loneliness, problematic smartphone use provides alternative stimulation. Individuals engage in behaviors such as watching short videos, using WeChat, and browsing websites, immersing themselves in virtual worlds to achieve emotional displacement. Within China’s digital ecosystem, platforms such as TikTok, Weibo, and Xiaohongshu provide abundant instant entertainment content, making this avoidance mechanism more convenient and appealing. Consequently, the stronger the feelings of loneliness, the more frequently individuals engage in repetitive smartphone use for emotional avoidance.

**Hypothesis** **4.** *Loneliness will be positively associated with problematic mobile phone use.*

### 1.4. The Moderating Effect of Peer Support

Based on social affiliation theory, when university students interact with artificial intelligence, their social needs are not genuinely satisfied, leading to heightened need for affiliation and increased prosocial behavior. However, this theoretical mechanism may exhibit differential manifestations across various social contexts. Social affiliation theory emphasizes that individuals’ social quality monitoring systems comprehensively assess the overall social environment rather than focusing solely on single interaction sources. For university students experiencing campus life and abundant peer interaction opportunities, peer support serves as crucial social signal input, influencing their internal monitoring systems’ judgments regarding social adequacy (Chai et al., 2021). This mechanism may be more pronounced within China’s collectivistic cultural background, as Chinese university students’ social identity and psychological well-being depend more heavily on group support and collective need for affiliation.

From the perspective of social affiliation theory’s monitoring mechanisms, peer support directly affects individuals’ social quality assessment processes, moderating the impact intensity of AI interaction frequency on the need for affiliation (Baumeister & Leary, 1995). Specifically, when university students possess adequate peer support, their social quality monitoring systems can receive rich social signals from authentic interpersonal interactions, such as social approval and emotional resonance. These reliable social experiences from peers provide the monitoring system with baseline references for social adequacy, ensuring that the lack of social cues in AI interactions does not trigger intense “social insufficiency” alerts, thereby generating a lower need for affiliation.

Within China’s collectivistic cultural environment on university campuses, this “social buffering effect” may be more pronounced. When university students can obtain sufficient emotional support and social recognition from roommates, classmates, or study groups, they demonstrate higher tolerance for the missing interpersonal warmth and emotional resonance in AI interactions. This cultural adaptive mechanism enables individuals with high peer support to view AI tools as purely functional assistance without generating expectations or disappointments regarding social substitution.

Conversely, under low-peer-support circumstances, individuals’ social monitoring systems frequently operate at socially insufficient baseline levels (Qualter et al., 2015). In this context, the dehumanizing characteristics of AI interactions become more prominent, and monitoring systems more readily identify this interaction pattern of lacking authentic emotional feedback as social threat signals. This sensitization effect causes social monitoring systems to overreact to AI interactions, interpreting originally neutral technological interactions as more intense social-exclusion experiences, thereby triggering a more urgent need for affiliation and stronger compensatory behavioral impulses. For Chinese university students studying away from home, lacking intimate friendships, or facing social difficulties, this mechanism may lead them to develop unrealistic social expectations toward AI interactions. When expectations are unmet, it instead exacerbates their social anxiety and desire for belongingness.

**Hypothesis** **5.** *Peer support moderates the relationship between AI interaction frequency and prosocial behavior through the need for affiliation. Under high-peer-support, this positive relationship weakens; under low-peer-support conditions, this positive relationship strengthens.*

Beyond moderating the need for affiliation pathway, peer support also moderates the process through which AI interaction frequency influences problematic smartphone use via loneliness. In this mechanism, authentic social connections provide genuine emotional feedback and nonverbal signals that AI systems cannot offer, satisfying individuals’ needs for personalized, empathically resonant interpersonal communication (Thoits, 1985). Such connections reduce the gap between individuals’ perceived expected social connection levels and actual obtained levels.

Under high-peer-support circumstances, social threats from AI interactions are alleviated, loneliness weakens, and individuals’ needs for emotional avoidance through problematic smartphone use correspondingly decrease. Particularly within China’s university dormitory culture, daily companionship, academic mutual assistance, and emotional exchanges among roommates provide individuals with continuous social connection. This “social safety net” ensures that the coldness and mechanistic nature of AI interactions do not trigger intense emotional deprivation experiences. Even with frequent AI tool usage, these students do not therefore experience significant loneliness and naturally do not need to seek emotional comfort through excessive smartphone use.

Conversely, under low-peer-support circumstances, the gap between expected and actual social connection levels significantly increases, loneliness intensifies, and problematic smartphone use increases. For university students lacking intimate peer relationships, AI interactions may become their primary “social” method, but such pseudo-social experiences may instead intensify their longing for authentic interpersonal connections. When this longing cannot be satisfied, they more readily turn to virtual worlds in smartphones seeking immediate emotional fulfillment.

**Hypothesis** **6.** *Peer support moderates the relationship between AI interaction frequency and problematic smartphone use through loneliness. Under high-peer-support conditions, this positive relationship weakens; under low-peer-support conditions, this positive relationship strengthens.*

## 2. Methods

### 2.1. Participants and Procedure

In May 2025, a longitudinal study was conducted among undergraduate students from the first to fourth year at institutions including Anyang University of China and Henan Normal University. All procedures adhered to the ethical principles for medical research involving human subjects outlined in the Declaration of Helsinki, as well as other applicable laws, rules, and ethical codes. To ensure the integrity of the study, all participating investigators underwent rigorous training prior to the survey administration. University administrators granted permission to contact students through official class WeChat groups. A research assistant then posted a recruitment notice in these groups to outline the study’s purpose, time commitment, and confidentiality assurances. The notice included a link to an online informed consent form and the hosted questionnaire. After receiving informed consent from the students, an electronic questionnaire was disseminated via the WeChat platform. Participants’ familiarity with AI tools was assessed through a multi-item screening questionnaire administered prior to the formal study. Furthermore, they were asked to indicate their frequency of use on a 5-point Likert scale (1 = less than once a month, 5 = multiple times a day) and to estimate their total duration of experience in months. Only participants who reported both a frequency of use of ‘3’ or higher and a total experience exceeding 6 months were included in the final sample.

This study is grounded in O’Connor and Rosenblood’s (1996) theory, which posits that humans’ regulation of social interaction needs exhibits immediate characteristics. When social needs are not adequately satisfied (e.g., through artificial intelligence interactions substituting for interpersonal interactions), individuals rapidly generate psychological responses, including an increased need of belongingness and heightened loneliness. This immediate response mechanism renders short-term longitudinal studies an ideal research approach. Furthermore, university students engage in frequent daily interactions, making a one-week interval sufficient to capture adequate behavioral variation. Consequently, a one-week period was established as the measurement node for this study. Moreover, previous studies have measured loneliness and the need for affiliation using a one-week interval (Tang et al., 2023), which can provide valuable methodological reference for the current research.

To ensure data quality, responses meeting the following criteria were excluded: (1) samples with missing values; (2) responses exhibiting homogeneity and regularity (e.g., nearly identical answers across all questions); (3) responses that were too short (e.g., less than 60 s). At Time 1, researchers distributed 450 questionnaires, of which 432 were deemed usable, yielding a 96% response rate. At Time 2, questionnaires were distributed to those who had completed the Time 1 survey, and 413 usable questionnaires were returned, achieving a 95.60% response rate. At Time 3, questionnaires were distributed to participants who had completed both Time 1 and Time 2 surveys, resulting in 406 usable questionnaires and a 98.31% response rate. After matching the questionnaires obtained from all three waves, a final sample of 388 usable questionnaires was included in the analysis, yielding an overall response rate of 86.22%. The mean age of participants was 19.708 years (SD = 1.118), ranging from 17 to 23 years. The sample comprised 160 boys (41.237%) and 228 girls (58.762%). Regarding geographic origin, 150 participants were from urban areas (38.659%) and 238 were from rural areas (61.34%). The distribution by academic year was as follows: first-year students constituted 196 participants (50.52%), second-year students 134 (34.53%), third-year students 40 (10.31%), and fourth-year students 18 (4.64%).

### 2.2. Measures

All constructs were assessed by using well-established and validated scales. Without further explanation, measures were translated into participants’ native language using recommended back-translation procedures.

#### 2.2.1. AI Interaction Frequency at Time 1

AI interaction frequency was measured using five items revised by Tang et al. (2023), based on the original scale developed by Shi et al. (2013). Previous research has demonstrated good internal consistency for this scale (Cronbach’s α = 0.93). Sample items include “How frequently do you interact with AI for work- and study-related purposes?” and “How often do you use AI to handle work and study matters?” Responses were measured on a 5-point Likert scale. Reliability and validity were confirmed in a Chinese sample through a pilot study. Cronbach’s alpha for the scale in this study was 0.886.

#### 2.2.2. Peer Support at Time 1

We assessed peer support using the four-item peer support subscale from the Chinese version of the Multidimensional Scale of Perceived Social Support, as revised by Yang et al. (2024). Sample items include “My friends can truly help me” and “I can rely on my friends when difficulties arise.” Responses were measured on a 7-point Likert scale. The subscale demonstrated excellent internal consistency in the current sample, with a Cronbach’s α of 0.900.

#### 2.2.3. Need for Affiliation at Time 2

Need for affiliation was measured using three items revised by Tang et al. (2023), based on the original scale developed by Wiesenfeld et al. (2001). Previous research has demonstrated good internal consistency for this scale (Cronbach’s α = 0.82). Sample items are “I think being close to others, listening to them, and relating to them is one of my favorite and most satisfying pastimes,” and “would find it very satisfying to be able to form new friendships with whomever I liked.” Responses were measured on a 7-point Likert scale. Cronbach’s alpha across observations for the scale was 0.867.

#### 2.2.4. Loneliness at Time 2

Loneliness was measured using three items revised by Tang et al. (2023), based on the original scale developed by Gabriel et al. (2021). Previous research has demonstrated good internal consistency for this scale (Cronbach’s α = 0.84). Sample items are “I lack companionship from one or more classmates,” “I feel neglected by one or more classmates,” and “I feel isolated by one or more classmates.” Responses were measured on a 5-point Likert scale. Cronbach’s alpha across observations for the scale was 0.843.

#### 2.2.5. Problematic Mobile Phone Use at Time 2

This study used the 10-item concise version of the Mobile Phone Problem Use Scale, which was modified by Foerster et al. (2015) and keeps good reliability and validity with the original full version developed by Bianchi and Phillips in 2005 (Bianchi & Phillips, 2005), which used a 5-point Likert scale for assessment. The scale used in this study is a single-factor model. Previous research has supported the robust validity and reliability of the Chinese version of the Mobile Phone Problem Use Scale among Chinese adolescents (Yao et al., 2022). The 10-item short form of the Mobile Phone Problem Use Scale (MPPUS-10) was indeed validated in a Chinese undergraduate sample by Yuan et al. (2023). Sample items are “When I’m in a bad mood, I use my mobile phone to make myself feel better” and “I find that I spend more time on my mobile phone than I intended”. Cronbach’s alpha for the scale used in this study was 0.920.

#### 2.2.6. Prosocial Behavior at Time 3

Prosocial behavior was measured using a three-item version as revised by Tang et al. (2023), based on the original scale developed by Yue et al. (2017). Previous research has demonstrated good internal consistency for this scale (Cronbach’s α = 0.88). Sample items include “Help other students when their workload is obviously excessive,” “Extend a helping hand to classmates when needed,” and “Assist other students in meeting their academic needs.” Responses were measured on a 5-point Likert scale. Cronbach’s alpha across observations for the scale was 0.781.

### 2.3. Analysis of Data

First, Harman’s one-factor test was conducted to examine potential common method bias. Second, descriptive statistics (means and standard deviations) and Pearson correlations were calculated for all study variables. Third, mediation and moderated mediation analyses were employed to test the hypothesized models, with demographic variables (age, gender, place of residence, and grade) included as controls. We used the default maximum likelihood estimator in Mplus 8.3 and tested all hypotheses simultaneously. Mediation and moderated mediation effects were examined using a parametric bootstrap procedure with 20,000 replications to generate 95% bias-corrected confidence intervals (CIs; Preacher et al., 2010; Selig & Preacher, 2009). Significance was determined by *p* < 0.05 or when the 95% CIs did not include zero.

## 3. Results

### 3.1. Common Method Bias Test

We conducted Harman’s one-factor test to examine the potential issue of common method bias. The results identified seven factors, with the first factor accounting for 25.59% of the variance, which is below the critical threshold of 40% (Podsakoff et al., 2003). Common method bias is therefore not a major concern in this study.

### 3.2. Preliminary Analysis

Table 1 presents descriptive statistics and Pearson correlations for the study variables.

### 3.3. Testing the Mediation Model

**Figure 2:**
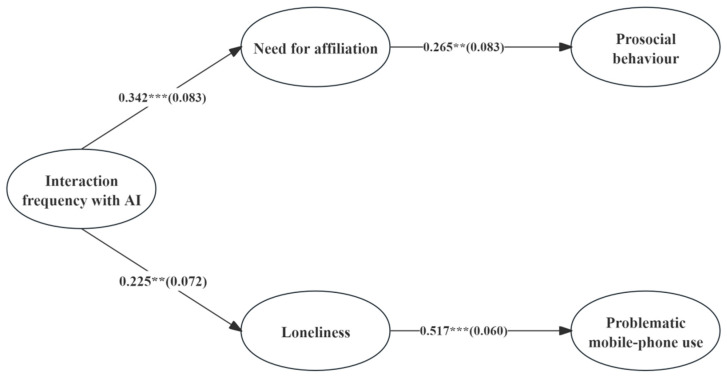
The results of testing the mediating model. (** *p* < 0.01,*** *p* < 0.001).

To examine the relationship between AI interaction frequency, the need for affiliation, loneliness, and problematic mobile phone use, we propose the following hypotheses: H1: AI interaction frequency will be positively associated with the need for affiliation; H2: the need for affiliation will be positively associated with prosocial behavior; H3: AI interaction frequency will be positively associated with problematic mobile phone use; H4: loneliness will be positively associated with problematic mobile phone use. We tested the hypothesized mediation models while controlling for demographic variables (age, gender, place of residence, and grade). The structural equation modeling results are shown in Figure 2 (for brevity, demographic control paths are not presented). AI interaction frequency significantly and positively predicted need for affiliation (β = 0.342, *p* < 0.001), supporting Hypothesis 1. The need for affiliation, in turn, significantly and positively predicted prosocial behavior (β = 0.342, *p* = 0.001), supporting Hypothesis 2. The need for affiliation therefore mediated the relationship between AI interaction frequency and prosocial behavior. The indirect effect was 0.091 (*SE* = 0.032, 95% CI = [0.039, 0.116]).

Moreover, AI interaction frequency significantly and positively predicted loneliness (β = 0.225, *p* = 0.002), supporting Hypothesis 3. Loneliness, in turn, significantly and positively predicted problematic mobile phone use (β = 0.517, *p* < 0.001), supporting Hypothesis 4. Loneliness therefore mediated the relationship between AI interaction frequency and problematic mobile phone use. The indirect effect was 0.116 (*SE* = 0.043, 95% CI = [0.040, 0.206]).

### 3.4. Testing the Moderated Mediation Model

The results of testing the moderated mediation model are presented in Table 2. In Model 1, the mediating role of the need for affiliation in the relationship between AI interaction frequency and prosocial behavior remained significant. In addition, the interaction term between AI interaction frequency and peer support significantly and negatively predicted the need for affiliation (β = −0.104, *SE* = 0.051, 95% CI [−0.205, −0.004]).

**Figure 3:**
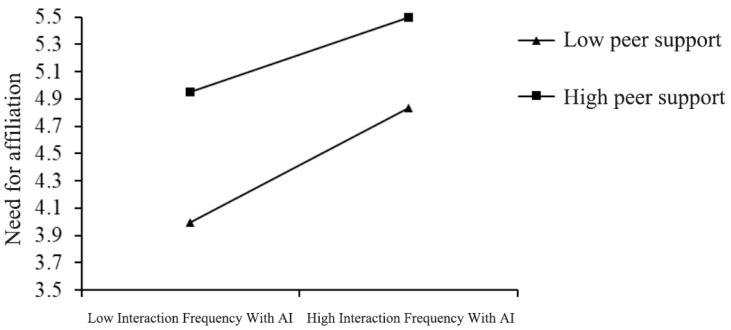
Peer support moderates the relationship between AI interaction frequency and need for affiliation.

To further examine the moderation direction, we conducted a simple slope analysis (see Figure 3). The relationship between AI interaction frequency and the need for affiliation was stronger at lower levels of peer support (−1 SD; B = 0.791, *p* < 0.001) compared to higher levels of peer support (+1 SD; B = 0.426, *p* < 0.001). The difference between the two slopes was also significant (difference = −0.365, *p* < 0.05). Hypothesis 5 was therefore supported.

In Model 2, the mediating role of loneliness in the relationship between AI interaction frequency and problematic mobile phone use remained significant. However, the interaction term between AI interaction frequency and peer support did not significantly predict loneliness (β = −0.086, *SE* = 0.055, 95% CI [−0.194, 0.022]), and Hypothesis 6 was thus not supported.

## 4. Discussion

Based on social affiliation theory, this study explores the mechanisms and boundary conditions through which university students’ AI interaction frequency influences their adaptive behaviors within the Chinese higher education context. The study employed a multi-wave longitudinal design, considering the time-sensitive nature of relevant variables, and collected data on a weekly basis to capture behavioral changes resulting from university students’ collaboration with artificial intelligence.

Empirical analysis revealed that AI interaction frequency was positively correlated with both the need for affiliation and loneliness, translating into increased prosocial behavior and increased problematic smartphone use, respectively. Peer support level moderated these relationships: under high-peer-support conditions, the influence of AI interaction frequency on prosocial behavior through the need for affiliation weakened; under low-peer-support conditions, this influence strengthened. Contrary to our hypothesis, peer support did not significantly moderate the indirect relationship between AI interaction frequency and problematic mobile phone use through loneliness.

### 4.1. Theoretical Significance

Our research makes several important contributions. Firstly, previous research on the integration of AI into college students has focused on either its benefits or its drawbacks, rarely considering both aspects simultaneously. Based on the social affiliation theory framework, this study constructs a dual-path model that explores how AI interaction frequency influences college students’ adaptive behaviors, revealing AI interaction as a new form of interpersonal interaction. Frequent interaction with AI is associated with both the need for affiliation and feelings of loneliness and is linked to two different behavioral outcomes: prosocial behaviors and problematic mobile phone use. In connection with these two states, college students exhibit both helping behaviors and problematic mobile phone use. It is noteworthy that prosocial behavior and problematic smartphone use are not mutually exclusive; rather, they represent two “co-occurring outcomes” stemming from frequent interactions with artificial intelligence among university students. This provides support for Chinese college students to develop healthy social skills and technology engagement in the age of AI and offers a reference for examining the social affiliation theory across different cultural contexts.

Secondly, by examining the relationships between the variables, we found that although AI interaction frequency can simultaneously generate the need for belonging and loneliness, and subsequently adaptive behaviors, the correlation between AI interaction frequency and the need for affiliation is stronger than that between AI interaction frequency and loneliness; meanwhile, loneliness is more likely to trigger problematic behaviors (such as problematic mobile phone use), whereas the behavioral effects of the need for affiliation in eliciting prosocial behaviors (such as altruistic behavior) are relatively limited. Based on this, we speculate that the need for affiliation promptly elicits a desire for authentic social bonds, which constitutes a relatively direct and proactive psychological response. In contrast, loneliness emerges as a passive emotional experience when the social monitoring system consistently detects a lack of meaningful social interaction; rather than being an immediate reaction, loneliness arises as a subsequent response to perceived social deficiency. While the need for affiliation reflects a motivational state driving active social seeking and can be partially satisfied through relatively straightforward social cues, loneliness entails more intricate emotional and cognitive components. The correlation between AI interaction frequency and the need for affiliation is therefore stronger than that between AI interaction frequency and loneliness. Additionally, compared to prosocial behaviors that arise to satisfy the need for affiliation, problematic mobile phone use is a passive, convenient way to escape reality. College students can use their phones to browse the web or watch short videos anywhere, anytime, to avoid loneliness; in contrast, prosocial behaviors, as an active action, require individuals to expend more resources (DeWall et al., 2008). When excessive interaction with artificial intelligence leads to feelings of loneliness among university students, it is important to recognize the inherent complexity of loneliness itself and acknowledge the gap between this sense of loneliness and their expectations of reality. In responding to loneliness, caution should be exercised in mobile phone use to prevent overreliance.

Thirdly, this study provides an important theoretical supplement to the antecedent research on core variables such as the need for affiliation, loneliness, prosocial behaviors, and problematic mobile phone use. In the current era of rapid AI technology diffusion, how individuals maintain a healthy social adaptation model in a human–machine coexisting environment has become a cutting-edge issue in psychology and information systems research. To our knowledge, existing studies on the antecedents of social behavior mainly focus on the influence mechanisms of external environments, such as family and peers (Mestre et al., 2019) and social media (M. Liu et al., 2024), and personal traits such as intelligence (Oda et al., 2014) and creativity (Kotaman et al., 2024). However, the role of technological factors, particularly the frequency of AI interaction in the context of higher education’s digital transformation, remains largely unexplored (Crompton & Burke, 2023). Our research indicates that greater participation in AI interaction is significantly associated with college students’ need for affiliation, which, in turn, promotes prosocial behavior. This finding suggests that AI interaction, as a product of higher education’s digital transformation, may represent an important technological factor associated with college students’ need for affiliation and helping behaviors. Similarly, this study also found that an increase in AI interaction frequency exhibited a significant positive association with individual’s loneliness, which was further correlated with increased problematic mobile phone use. In this regard, our work is one of the few to examine the impact of AI interaction frequency on college students’ social behaviors, thereby paving the way for future research further exploring how interactions with AI are related to their helping behaviors and problematic mobile phone use, and to inform effective suggestions for alleviating the loneliness associated with AI interactions.

Finally, our study offers a theoretical extension to social affiliation theory model by emphasizing the moderating role of peer support in the relationship between AI interaction frequency, the need for affiliation, and college students’ prosocial behaviors. This extends the application scope of the model to the human–AI interaction domain. These findings suggest the potential applicability of the model in the context of human–AI interaction, providing a preliminary framework for understanding the dual effects of college students’ engagements with AI. Specifically, while the mediating mechanism by which AI interaction frequency stimulates the need for belonging and thus promotes prosocial behavior is universal, the extent of its influence shows significant individual differences. Our study indicates that peer support could serve as an important boundary condition, potentially attenuating the effects of AI interaction on college students, which holds important theoretical significance. The study found that high levels of peer support can effectively compensate for the emotional connection deficits caused by AI interactions, thereby weakening the intensity of an individual’s need for belonging and correspondingly reducing prosocial behavior motivated by the need for belonging (Baumeister & Leary, 1995; Mou & Xu, 2017; Naslund et al., 2016). It is noteworthy that the moderating effect of peer support was not verified in the “AI interaction frequency influencing problematic mobile phone use through loneliness” mediating path. Regarding this, the reason for the lack of significance may lie in the nature of the loneliness generated after interacting with AI—it is essentially an uncomfortable emotional state, a pain arising from the gap between the desire for social connection and the reality of its absence (Russell et al., 1980). To alleviate this pain, an individual’s most direct reaction is to “escape” this discomfort (Lazarus & Folkman, 1984). A mobile phone allows one to quickly shift attention away from inner emptiness and discomfort into a virtual world of information overload and constant novel stimulation (H. Wang et al., 2025). The mechanisms of social support and loneliness differ fundamentally, which may pose challenges for peer support in effectively competing with this avoidance mechanism. In summary, this study highlights the contextual dependency of college students’ AI interaction frequency and the importance of peer support, offering detailed insights into shaping the mechanisms and conditions of college students’ interactions with AI.

### 4.2. Practical Significance

Our research provides valuable insights for shaping how higher education can cultivate college students and aid their personal development. Firstly, it profoundly reveals the complex dual effects mechanism generated by AI interaction frequency. College students should therefore adopt metacognitive regulation strategies during AI use, fully recognizing that frequent AI interactions not only offer high efficiency and convenience but also carry potential costs such as a lack of belongingness and an increase in feelings of loneliness, thereby achieving a scientific and rational use of AI.

Secondly, based on the finding that an excessive frequency of AI interaction is associated with an increased need for affiliation among university students, rationally controlling the density of AI in the learning environment is recommended. University students should consciously evaluate the frequency and extent of their reliance on AI tools in their daily studies. Over-dependence on ChatGPT, learning assistants, or other AI systems may weaken direct academic interaction with peers and instructors. Students need to strike a balance between leveraging the efficiency brought by AI and maintaining interpersonal academic engagement (Zhang et al., 2024; Klimova & Pikhart, 2025). This means that, while using AI for completing assignments, preparing for exams, or conducting research brings significant value, opportunities for discussing issues with classmates, participating in class interactions, and seeking guidance from teachers should still be preserved. It is also vital to consider that students with high-frequency AI interaction are more likely to engage in prosocial behavior to fulfill their need for affiliation. In addition, it is essential to rationally examine changes in helping behavior under the influence of AI. When university students find themselves more willing to help others after frequently using AI learning tools, they should carefully reflect on the true motivation behind this change. This increased tendency to help may not purely stem from altruism but could instead be a compensatory response to the lack of genuine social feedback in AI interactions. To maintain positive interpersonal relationships, students should therefore develop truly intrinsic motivations for helping—making such behavior more authentic and meaningful. Furthermore, university students need to pay special attention to whether the sense of loneliness triggered by AI interactions leads to problematic mobile phone use. When experiencing social emptiness after interacting with AI systems, students may seek immediate stimulation and virtual social fulfillment through mindlessly scrolling short videos, browsing social media, or immersing themselves in mobile games (Russell et al., 1980; Lazarus & Folkman, 1984). However, such behavior only exacerbates social isolation, creating a vicious cycle. Students should establish self-monitoring mechanisms for phone use, set reasonable time limits, and actively seek real human connections when feeling lonely, rather than escaping into the virtual world (H. Wang et al., 2025).

Thirdly, we found that for college students with high levels of peer support, the positive impact of AI interaction frequency on the need for affiliation and subsequent prosocial behaviors is weaker; conversely, the moderating effect becomes stronger. It is worth noting that although prosocial behaviors appear to be positive prosocial actions on the surface, their deeper psychological mechanisms essentially reflect the psychological compensation mechanism activated by individuals to fulfill their need for affiliation. Such compensatory helping behaviors may lack intrinsic motivation, and their sustainability and psychological health benefits warrant further exploration. This suggests that universities should offer specialized digital literacy courses, using case studies, experiential learning, and other methods, to help students deeply understand the technical limitations of AI interactions and establish a cognitive framework of “AI as a tool, not a partner”. For students with low levels of peer support, universities should create intensive interpersonal interaction platforms, such as small study groups (6–8 people), weekly face-to-face communication activities, and one-on-one peer mentor pairings, to meet their belonging needs through high-quality real-world social connections. Additionally, universities should establish dynamic assessment mechanisms to regularly track students’ social connection quality and the extent to which their belonging needs are met and adjust the intensity of support strategies in a timely manner.

### 4.3. Research Limitations

This study has several limitations, which pave the way for future research. Firstly, we adopted a longitudinal design to capture the dynamics of college students’ interactions with AI, thereby alleviating concerns about common method bias and enhancing ecological validity (e.g., Gabriel et al., 2019; Heggestad et al., 2022). Its relatively short timeframe and our reliance on a survey-based approach, primarily utilizing self-report measures, may limit our ability to draw conclusions and could introduce potential biases associated with self-reported data. While our three-wave design is an improvement over cross-sectional studies, the one-week intervals between measurements still represent a relatively short timeframe for capturing the long-term evolution of the psychological constructs we examined (e.g., loneliness, the need for affiliation). We note that longer-term longitudinal studies are needed to observe more profound developmental trajectories. Future research could address this issue by using experimental designs or mixed methods, combining qualitative and quantitative data to replicate and extend our findings. These methods could enhance internal validity through stronger causal inference and improve external validity by robustly and comprehensively testing our theory (Lu et al., 2024). Additionally, our sample comes exclusively from Chinese college students, providing a concentrated context for studying these phenomena but inherently limiting the generalizability of our findings to other cultural contexts. Conducting cross-cultural studies to generalize the results and explore the potential cultural contingencies of college students’ AI interactions would be a productive direction for future research.

Secondly, although we identified the need for affiliation and loneliness as key psychological mechanisms linking college students’ AI interactions with prosocial behaviors and problematic mobile phone use, the complexity of AI interactions means that other important mediating pathways remain to be explored. The potential influence of other cognitive mechanisms remains unclear. Future research could therefore draw on cognitive theories, such as Cognitive Load Theory or Creative Cognition Theory, to explore whether AI interactions might trigger other pathways—for instance, fostering cognitive dependency or stimulating creative thinking. Future research should therefore adopt an integrated theoretical framework to systematically explore the multi-level cognitive, emotional, and behavioral effects of AI interaction.

This study did not control for certain variables. Digital literacy (Tinmaz et al., 2022), as an individual difference variable, may affect the psychological effects of AI interaction. Individuals with higher digital literacy possess stronger critical thinking abilities and media awareness, allowing them to more accurately identify and assess the pros and cons of AI interactions, leading to more rational and adaptive usage patterns. Additionally, academic pressure, as a situational factor, may influence an individual’s motivation for technology use through the stress-coping theory mechanism.

Finally, we believe that prosocial behaviors and problematic mobile phone use may jointly predict a negative side effect: the prosocial behaviors that college students engage in to fulfill their need for affiliation are not necessarily long-term effective social skills. Instead, they may serve merely to compensate for their need for affiliation. While prosocial behavior may help university students gain positive experiences in the short term, the nature and long-term outcomes of these behaviors warrant further investigation. Future research thus still needs to adopt more rigorous methodologies, such as experimental approaches, to focus on the subsequent effects of artificial intelligence on university students’ prosocial behaviors.

It is noteworthy that our study is based on surveys conducted with Chinese college student samples, and the conclusions of this research are therefore more applicable to that specific cohort. If broader generalization is needed, cross-cultural validation and experimental studies are required.

## Figures and Tables

**Table 1:** Descriptive statistics and correlations among study variables.

	M	SD	1	2	3	4	5	6
1 = AI interaction frequency	3.169	0.600	(0.862)					
2 = Need for affiliation	4.863	0.918	0.313 **	(0.868)				
3 = Peer support	5.151	0.830	0.033	0.449 **	(0.902)			
4 = Loneliness	1.870	0.665	0.205 **	0.010	−0.219 **	(0.833)		
5 = Problematic mobile phone use	2.752	0.699	0.325 **	0.215 **	−0.059	0.452 **	(0.905)	
6 = Prosocial behavior	3.934	0.403	−0.051	0.191 **	0.186 **	−0.048	−0.094	(0.782)

*Note*. (*N* = 388). Scale reliabilities are reported along the diagonal in parentheses. ** *p* < 0.01.

**Table 2:** The results of testing the moderated mediating model.

Model 1	Need for Affiliation			Prosocial Behavior		
	β	SE	95%CI	β	SE	95%CI
Age	−0.019	0.054	[−0.125, 0.087]	0.037	0.065	[−0.09, 0.164]
Gender	−0.043	0.054	[−0.149, 0.062]	−0.057	0.065	[−0.184, 0.07]
Place of residence	−0.022	0.054	[−0.127, 0.083]	0.069	0.065	[−0.058, 0.196]
Grade	−0.008	0.054	[−0.115, 0.098]	0.063	0.065	[−0.063, 0.19]
AI interaction frequency	0.353 *	0.055	[0.245, 0.462]			
Need for affiliation				0.278	0.067	[0.148, 0.409]
Peer support	0.472 *	0.051	[0.371, 0.572]			
AI interaction frequency × Peer support	−0.104 *	0.051	[−0.205, −0.004]			
R2	0.371			0.092		
**Model 2**	**Loneliness**			**Problematic Mobile Phone Use**		
	**β**	**SE**	**95%CI**	**β**	**SE**	**95%CI**
Age	−0.021	0.06	[−0.139, 0.097]	0.001	0.055	[−0.106, 0.109]
Gender	−0.066	0.06	[−0.184, 0.052]	−0.069	0.055	[−0.177, 0.039]
Place of residence	0.006	0.06	[−0.111, 0.123]	0.055	0.055	[−0.053, 0.162]
Grade	−0.116	0.06	[−0.233, 0.002]	0.056	0.055	[−0.052, 0.165]
AI interaction frequency	0.257	0.064	[0.131, 0.383]			
Loneliness				0.519	0.052	[0.418, 0.621]
Peer support	−0.256	0.061	[−0.375, −0.137]			
AI interaction frequency × Peer support	−0.086	0.055	[−0.194, 0.022]			
R2	0.153			0.280		

*N* = 388. Control variables include age, gender, place of residence, and grade. * *p* < 0.05.

## Data Availability

The data presented in this study are available on request from the corresponding author due to privacy restrictions.
